# Multilineage differentiation potential of hematoendothelial progenitors derived from human induced pluripotent stem cells

**DOI:** 10.1186/s13287-020-01997-w

**Published:** 2020-11-11

**Authors:** Ratchapong Netsrithong, Siriwal Suwanpitak, Bootsakorn Boonkaew, Kongtana Trakarnsanga, Lung-Ji Chang, Chartsiam Tipgomut, Chinnavuth Vatanashevanopakorn, Kovit Pattanapanyasat, Methichit Wattanapanitch

**Affiliations:** 1 Siriraj Center for Regenerative Medicine, Research Department, Faculty of Medicine Siriraj Hospital, Mahidol University, Bangkok, 10700 Thailand; 2 Department of Immunology, Faculty of Medicine Siriraj Hospital, Mahidol University, Bangkok, Thailand; 3 Department of Biochemistry, Faculty of Medicine Siriraj Hospital, Mahidol University, Bangkok, Thailand; 4 Shenzhen Genoimmune Medical Institute, Shenzhen, China; 5 Siriraj Center of Research Excellence for Microparticle and Exosome in Diseases, Research Department, Faculty of Medicine Siriraj Hospital, Mahidol University, Bangkok, Thailand

**Keywords:** Induced pluripotent stem cells, Hematopoietic differentiation, Hematoendothelial progenitors, Endothelial cells, T lymphocytes, Erythroid cells

## Abstract

**Background:**

Human induced pluripotent stem cells (hiPSCs) offer a renewable source of cells for the generation of hematopoietic cells for cell-based therapy, disease modeling, and drug screening. However, current serum/feeder-free differentiation protocols rely on the use of various cytokines, which makes the process very costly or the generation of embryoid bodies (EBs), which are labor-intensive and can cause heterogeneity during differentiation. Here, we report a simple feeder and serum-free monolayer protocol for efficient generation of iPSC-derived multipotent hematoendothelial progenitors (HEPs), which can further differentiate into endothelial and hematopoietic cells including erythroid and T lineages.

**Methods:**

Formation of HEPs from iPSCs was initiated by inhibition of GSK3 signaling for 2 days followed by the addition of VEGF and FGF2 for 3 days. The HEPs were further induced toward mature endothelial cells (ECs) in an angiogenic condition and toward T cells by co-culturing with OP9-DL1 feeder cells. Endothelial-to-hematopoietic transition (EHT) of the HEPs was further promoted by supplementation with the TGF-β signaling inhibitor. Erythroid differentiation was performed by culturing the hematopoietic stem/progenitor cells (HSPCs) in a three-stage erythroid liquid culture system.

**Results:**

Our protocol significantly enhanced the number of KDR^+^ CD34^+^ CD31^+^ HEPs on day 5 of differentiation. Further culture of HEPs in angiogenic conditions promoted the formation of mature ECs, which expressed CD34, CD31, CD144, vWF, and ICAM-1, and could exhibit the formation of vascular-like network and acetylated low-density lipoprotein (Ac-LDL) uptake. In addition, the HEPs were differentiated into CD8^+^ T lymphocytes, which could be expanded up to 34-fold upon TCR stimulation. Inhibition of TGF-β signaling at the HEP stage promoted EHT and yielded a large number of HSPCs expressing CD34 and CD43. Upon erythroid differentiation, these HSPCs were expanded up to 40-fold and displayed morphological changes following stages of erythroid development.

**Conclusion:**

This protocol offers an efficient and simple approach for the generation of multipotent HEPs and could be adapted to generate desired blood cells in large numbers for applications in basic research including developmental study, disease modeling, and drug screening as well as in regenerative medicine.

## Background

Human induced pluripotent stem cells (hiPSCs) have unlimited proliferation and ability to differentiate into all mature cell types [1]. Therefore, hiPSCs have become a potential renewable source of cells for the generation of a clinically relevant number of hematopoietic stem/progenitor cells (HSPCs) for cell-based therapy. Although many studies were able to generate various hematopoietic cell types from hiPSCs [2–5], efficient production of adult-type HSPCs with robust engraftment potential and their derivatives including mature functional erythrocytes, megakaryocytes, and T cells remain significant challenges due to the complexity of the embryonic hematopoietic system. In addition, lack of stage-specific markers makes it difficult to identify different stages of blood cell development [6].

During embryo development, hematopoietic stem cells (HSCs) originated from the mesodermal germ layer are classified into two successive waves. These HSCs exhibit distinct differentiation potential and are specified at different periods during development. The early wave of hematopoiesis appears in the yolk sac and is described as primitive hematopoiesis. Primitive HSCs can give rise to primitive macrophages, megakaryocytes, and nucleated erythroblasts, which express embryonic hemoglobin, but not B and T lymphocytes [7, 8]. The second wave of hematopoiesis is definitive hematopoiesis, which is developed in the dorsal aorta of the aorta-gonad-mesonephros (AGM) region of the fetus, fetal liver and bone marrow [9]. The definitive hematopoiesis program produces HSCs with a long-term repopulating ability and the potential to generate myeloid cells, T lymphocytes, and enucleated erythrocytes, which express adult-type hemoglobin [10]. Efficient in vitro differentiation of HSPCs from human pluripotent stem cells (hPSCs) requires an understanding of signaling pathways that govern the early stages of hematopoiesis. The initial formation of mesodermal cells requires bone morphogenetic protein 4 (BMP4) signaling [11, 12]. During differentiation, hPSCs develop to mesodermal cells that express mesodermal markers such as apelin receptor (APLNR), platelet-derived growth factor receptor (PDGFR)α/CD140a, KDR, Brachyury (T), MIXL1, FOXF1, and GATA2 [13–15]. Modulation of activin-nodal and Wnt-β-catenin signaling pathways leads to the formation of primitive or definitive HSCs. Primitive progenitors (KDR^+^CD235a^+^) require activin-nodal signaling and inhibition of the Wnt-β-catenin signaling pathway. In contrast, definitive progenitors (KDR^+^CD235a^−^) require Wnt-β-catenin signaling. The addition of a Wnt agonist, CHIR99021, during the mesodermal induction has been shown to promote the definitive hematopoiesis program but inhibit the primitive hematopoiesis [7]. Mesodermal cells can be specified further toward hematoendothelial progenitors or HEPs (CD144^+^CD34^+^CD31^+^) by the addition of key cytokines such as fibroblast growth factor 2 (FGF2) and vascular endothelial growth factor (VEGF) [16, 17]. Following in vitro differentiation, multipotent hematopoietic progenitors (CD34^+^CD43^+^ or CD34^+^CD45^+^) emerge from the HEPs through the process called endothelial-to-hematopoietic transition (EHT) [18, 19].

To date, there are two approaches to differentiate hPSCs toward HSPCs such as co-culturing hPSCs with murine bone marrow-derived feeder cells such as OP9 or MS5 cell line in the serum-containing medium [20, 21] and defined conditions with specific growth factors or cytokines via embryoid bodies (EBs) [7, 22] or monolayer system [23, 24]. The OP9 co-culture system is widely used to generate multipotent HSPCs, which can be differentiated further to several hematopoietic lineages including T lymphocytes [25, 26], B lymphocytes [27], megakaryocytes [28], macrophages [29, 30], and erythrocytes [3]. However, the efficiency of HSPC generation depends on the size of hPSC colonies, the density of OP9 cells, and fetal bovine serum (FBS) lot. Additionally, the use of murine stromal cell lines makes this system unsuitable for downstream therapeutic applications. On the other hand, the step-wise defined conditions with specific signals allow better recapitulation of embryonic hematopoiesis [15].

Previous studies demonstrated that the use of the GSK3 inhibitor CHIR99021, which activates the Wnt signaling pathway, is sufficient to induce HEPs and endothelial specification from hiPSCs [23, 31]. However, the EHT and differentiation potential of HEPs in these systems have not been investigated. In this study, we report a simple chemically defined system to generate multipotent HEPs from hiPSCs. The addition of CHIR99021 was sufficient to generate mesodermal cells (KDR^+^CD235a^−^) from hiPSCs within 3 days. These cells gave rise to HEPs, which were differentiated further to functional endothelial cells (ECs) upon angiogenic induction. On the other hand, inhibition of TGF-β signaling at the HEP stage led to efficient HSPC cluster formation from the HEP monolayer, marked by the expression of CD34 and CD43 with an ability to form all types of hematopoietic colonies in CFU assays. These HSPCs can be differentiated further toward erythroid and lymphoid lineages. The hiPSC-derived erythroblasts exhibited robust expansion and expression of hemoglobin, whereas the hiPSC-derived T lymphocytes expressed T cell markers, CD3, CD45, CD7, CD4, CD8, and TCRαβ. Further stimulation with anti-CD3/CD28, the hiPSC-derive T cells expanded up to 34-fold and expressed activation markers, CD107a, CD69, and CD25. Our study offers a simple and low-cost strategy to produce hiPSC-derived multipotent HEPs for the generation of ECs, HSPCs, erythroid cells, and T cells for basic research, including developmental study, disease modeling, and drug screening as well as applications in regenerative medicine.

## Methods

### Culture of iPSCs

We used the human iPSC lines generated in our laboratory from skin fibroblasts (MUSIi001-A) [32], peripheral blood T lymphocytes (MUSIi011-A) [33] and peripheral blood mononuclear cells (PBiPSC1). The use of PBMCs was approved by the Siriraj Institutional Review Board no. Si 404/2017 and Si146/2019, in accordance with the Helsinki declaration of 1975. Written informed consent was obtained from donors. Human iPSCs were maintained on Matrigel® (1 in 40 dilutions, Corning)-coated plates in Essential 8™ (E8) medium (Gibco) and subcultured every 5 days using 0.5 mM EDTA (Invitrogen) in phosphate buffered saline (PBS) without calcium and magnesium (Apsalagen) with a split ratio of 1 to 10.

### Differentiation of iPSCs toward HEPs and HSPCs

The monolayer induction protocol was performed following the previously published protocol [24] with modifications. Briefly, undifferentiated iPSCs were pre-treated with 10 μM Y-27632 (Selleckchem) prior to dissociation into single cells using TrypLE™ Select (Gibco) for 5 min at 37 °C. The cells were plated onto Matrigel® (1:40 dilutions)-coated culture plates at a density of 4.5 × 10^4^ cells/cm^2^ in E8 medium supplemented with 10 μM Y-27632. After 24 h, iPSCs were cultured in E8 medium for 2 days. On day 0, the medium was changed to the basal medium: RPMI1640 medium (Gibco), 2% B-27™ (Gibco), 2 mM GlutaMAX™ (Gibco), and 60 μg/mL ascorbic acid (Vit C, Sigma-Aldrich), supplemented with 6 μM GSK3 inhibitor CHIR990921 (Merck). On day 2, the medium was changed to the basal medium supplemented with 50 ng/mL VEGF (Peprotech) and 10 ng/mL FGF2 (Miltenyi Biotec). On day 3, cells were replated onto Matrigel®-coated culture plates using TrypLE™ Select at a density of 4 × 10^4^ cells/cm^2^ in the same medium. From days 0 to 4 of differentiation, the medium was changed every day. On days 5–8, the medium was replaced with the basal medium supplemented with 50 ng/mL VEGF, 10 ng/mL FGF2, and 10 μM TGFβ inhibitor SB431542 (Merck). On day 6, the medium was half changed. The adherent and floating cells were harvested at different time points for RNA extraction and flow cytometric analysis.

**Fig. 1:**
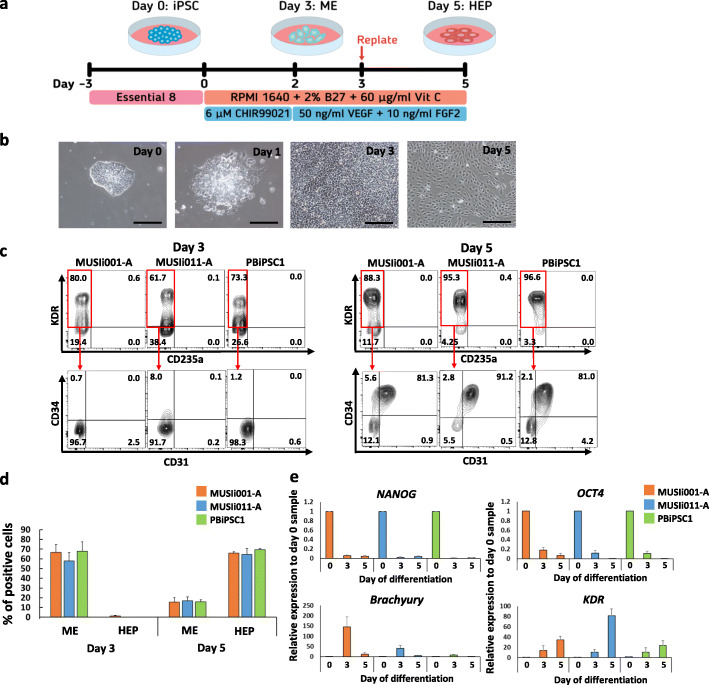
Differentiation of three human iPSC lines toward HEPs using the GSK3β inhibitor CHIR99021. **a** Schematic diagram of the differentiation protocol. The medium was changed daily during differentiation. **b** Representative images of differentiated MUSIi011-A cells from day 0 to 5 (scale bar = 200 μm). **c** Representative flow cytometric analysis shows the expression of HEP markers at day 3 and day 5 of differentiation. The KDR^+^CD235a^−^ cells were gated for analysis of HEP markers (KDR^+^CD34^+^CD31^+^). **d** Percentage of ME cells (KDR^+^CD235a^−^CD34^−^) and HEPs (KDR^+^CD34^+^CD31^+^) on day 3 and day 5. Data were obtained from at least three independent experiments (*N* ≥ 3). Error bars represent mean ± SEM. **e** Quantitative PCR analysis of pluripotent (*NANOG*, *OCT4*), mesendodermal (*Brachyury*), and mesodermal (*KDR*) genes at day 0, day 3, and day 5 of differentiation. Data were obtained from three independent experiments (*N* = 3). Error bars represent mean ± SEM

### Endothelial cell (EC) differentiation

On day 5, the differentiated cells were sorted using the CD34 MicroBead Kit (Miltenyi Biotec) according to the manufacturer’s instructions. The sorted CD34^+^ cells were seeded onto 0.1% gelatin (Sigma-Aldrich)-coated 6-well plates at a density of 1.5 × 10^5^ cells/well in endothelial cell growth medium (EGM; R&D Systems). The medium was changed every 2–3 days. Cells were cultured until reaching 80–90% confluence and passaged using TrypLE™ Select (Gibco) with a split ratio of 1 to 3.

Functional analyses of ECs were examined by in vitro tube formation of capillary structures and acetylated low-density lipoprotein (Ac-LDL) uptake. For tube formation assay, ECs were seeded onto Matrigel®-coated 96-well plates at a density of 1.5 × 10^4^ cells/well in EGM supplemented with 50 ng/mL VEGF. Human umbilical vein endothelial cells (HUVECs, ATCC® CRL-1730™) were used as a positive control and seeded at a density of 2 × 10^4^ cells/well. Time-lapse images were acquired every 20 min using an IncuCyte® Zoom Live-Cell Analysis System (Essen BioScience) over the 12-h period. The Ac-LDL uptake was performed by incubating the cells with 10 μg/mL Ac-LDL fluorescent probe 1,1′-dioctadecyl-3,3,3′,3′-tetramethyl-indocarbocyanine perchlorate (Dil-Ac-LDL) (Thermo Fisher Scientific) in EGM medium for 4 h at 37 °C. The cells were then washed with PBS and fixed with 4% paraformaldehyde (Sigma-Aldrich) for 10 min at room temperature. Nuclei were counterstained with DAPI. Images were acquired using the fluorescence microscope (Eclipse TiS, Nikon) and the NIS Elements D 4.20.00 64-bit software.

### Erythroid differentiation

Erythroid differentiation was performed using HSPCs that emerged from the adherent monolayer on day 8 of differentiation. The floating HSPCs were collected and transferred to a T25 flask for erythroid differentiation. We used the three-stage erythroid liquid culture following the previously published protocol [34]. Briefly, the HSPCs were cultured in an erythroid differentiation medium: IMDM containing 3% (v/v) human AB serum (Sigma-Aldrich), 2% (v/v) defined FBS (Hyclone), and 200 μg/mL transferrin (R&D Systems). On days 0–8 of differentiation (first stage), the medium was supplemented with 10 ng/mL stem cell factor (SCF), 1 ng/mL IL-3, and 3 U/mL EPO. On days 8–11 of differentiation (second stage), the medium was supplemented with 10 ng/mL SCF, 3 U/mL erythropoietin (EPO). In the last stage, the differentiated cells were cultured in the medium supplemented with 3 U/mL EPO and additional transferrin to a final concentration of 500 μg/mL until day 19.

### T lymphocyte differentiation

The generation of T lymphocytes was performed using the mouse stromal OP9-DL1 co-culture system. Briefly, 3 × 10^5^ OP9-DL1 cells were pre-seeded onto 0.1% gelatin-coated 10-cm culture dish in the OP9-DL1 medium: α-MEM medium (Gibco) containing 20% FBS (Merck), 2 mM GlutaMAX™ (Gibco), 100 U/mL Penicillin/Streptomycin (Gibco), and 100 μM 1-Thioglycerol (MTG; Sigma-Aldrich), and cultured for 2 days. T lymphocyte differentiation was performed according to the previous study with modifications [35]. Briefly, day 5 HEPs or day 8 floating HSPCs were harvested and transferred onto OP9-DL1 cells at a density of 1 × 10^6^ cells/dish in the OP9-DL1 medium supplemented with 10 ng/mL IL-7, 10 ng/mL FLT3L, and 10 ng/mL SCF (all from Biolegend). The medium was changed every 3 days. The differentiated cells were harvested and transferred onto the new OP9-DL1 cells every 6 days. After day 6 of T cell differentiation, the medium was changed to OP9-DL1 medium supplemented with 1 ng/mL IL-7 and 5 ng/mL FLT3L. On day 24, total floating cells were harvested and transferred to a well of 24-well plate at a density of 1 × 10^6^ cells/mL in RPMI medium supplemented with 10% FBS, 10 ng/mL IL-7, 10 ng/mL IL-15, and 500 μg/mL anti-CD3 antibody (Invitrogen) or 1% T Cell TransAct™ (Miltenyi Biotec) for 14 days. The differentiated cells were harvested at various time points for flow cytometric analyses of HSPC, T cell, and activation markers.

### Immunofluorescence staining

Cells were fixed with 4% paraformaldehyde for 15 min at room temperature. For intracellular staining, cells were permeabilized using 0.1% Triton X-100 in (PBS) for 10 min and blocked with 3% bovine serum albumin (BSA, Sigma-Aldrich) in PBS for 1 h at room temperature. The staining was performed using primary antibodies against CD31 (BioLegend, 1:50 dilution), ICAM-1 (CD54) (BioLegend, 1:50 dilution), VE-cadherin (CD144) (BioLegend, 1:50 dilution), and von Willebrand factor (vWF) (Thermo Fisher Scientific, 1:100 dilution) for 1 h at room temperature. After washing three times with PBS, cells were stained with secondary antibodies: anti-mouse antibody conjugated with Alexa Flour®-488 or Alexa Flour®-568 (Thermo Fisher Scientific) for 30 min at room temperature in the dark. Nuclei were counterstained with DAPI (Thermo Fisher Scientific). Fluorescence images were acquired using a fluorescence microscope (Eclipse TiS, Nikon) and the NIS Elements D 4.20.00 64-bit software.

### Reverse transcription and quantitative polymerase chain reaction

Total RNA was isolated using the Total RNA Purification Kit (GeneMark). Reverse transcription was performed using 1 μg of RNA and the RevertAid First Strand cDNA Synthesis Kit (Thermo Fisher Scientific) on the TProfessional Basic PCR Thermocycler (Biometra) according to the manufacturer’s instruction. Quantitative polymerase chain reaction (qPCR) was carried out on the CFX96™ Real-Time PCR detection system (Bio-Rad) using KAPA™ SYBR® FAST qPCR Master Mix Kit (Kapa Biosystems) and the primers listed in Additional file 1: Table S1. The cycle parameters were activation step at 95 °C for 1 min followed by 40 cycles of denaturation at 95 °C for 2 s, annealing at 60 °C for 30 s and extension at 70 °C for 2 s. The signal was acquired at the end of the annealing step. Data were normalized with a housekeeping gene, *GAPDH*, and expressions were plotted against the undifferentiated iPSCs. For erythroid-related genes, total RNA was isolated using Trizol reagent (Thermo Fisher Scientific) according to the manufacturer’s instruction. Four hundred nanograms of RNA was reverse transcribed into cDNA using SuperScript™ III reverse transcriptase (Thermo Fisher Scientific). RT-PCR was performed with the primers listed in Additional file 2: Table S2.

### Flow cytometry

For adherent cells, the cells were dissociated with TrypLE™ Select for 5 min and resuspended in FACS buffer (3% FBS in PBS). For floating cells, cells were harvested by gently pipetting without disturbing the adherent cell layers. The cells were blocked with 10% human AB serum in FACS buffer for 30 min at 4 °C followed by staining with fluorescently labeled antibodies for 15 min at room temperature. Antibodies for HEP and EC differentiation were CD34-Alexa Fluor® 700 (BioLegend), CD43-PE/Cy™7 (BioLegend), CD31-VioBlue® (Miltenyi Biotec), CD235a-PE (BioLegend), KDR (CD309)-APC (BioLegend), and CD144 (VE-cadherin)-FITC (Miltenyi Biotec). For erythroid differentiation, the cells were analyzed for the expression of early erythroid markers by staining with antibodies, CD36-FITC and CD49a-FITC, and late erythroid markers, GPA (CD235a) and Band 3 (IBGRL), followed by the secondary anti-mouse IgG-APC antibody (BioLegend). For analysis of T cell development and activation markers, the floating cells were harvested and stained with antibodies: CD3-FITC (BioLegend), CD45-PerCP (BioLegend), CD7-PE/Cy7 (BD Biosciences), CD4-Pacific Blue™ (BioLegend), CD8-Alexa Fluor® 700 (BioLegend), TCRαβ-APC/Cy™7 (BioLegend), CD25-PE (BioLegend), CD69-APC (BioLegend), and CD107a-APC/Cy™7 (BioLegend). The isotype-control antibodies used to determine non-specific background signal were APC-mouse IgG1, PE-mouse IgG1, PE-mouse IgG2a, FITC-mouse IgG1, VioBlue®-mouse IgG1, PerCP-mouse IgG1, Pacific Blue™-mouse IgG2b, APC/Cy™7-mouse IgG1, PE/Cy™7-mouse IgG1, and FITC-REA clone. Most antibodies were used at dilution 1:50 except for the primary anti-GPA and Band 3 antibodies, which were used at dilution 1:2. Zombie Aqua™ or Zombie Violet™ Fixable Viability Kit (BioLegend) was used to exclude dead cells. The compensation controls were prepared using BD™ CompBeads Set Anti-Mouse Ig (BD Biosciences). After labeling, cells were washed with FACS buffer and fixed with 1% paraformaldehyde at 4 °C. Flow cytometry analysis was performed using LSR-II (BD Biosciences), and the data were analyzed using FlowJo V10 software.

### Colony-forming unit (CFU) assay

Colony-forming unit (CFU) assay was performed using MethoCult™ H4434 Classic (Stemcell technologies) according to the manufacturer’s instruction. Briefly, 5 × 10^4^ of day 5 or day 8 differentiated cells were mixed with 4.4 mL of MethoCult™ medium and gently dispensed into two 35-mm dishes using 1-mL syringe. Cells were incubated at 37 °C, 5% CO_2_ with 95% humidity. Colony counting and classification were performed under a microscope on day 14 of culture.

### Cytospin and Leishman’s staining

Cultured cells were collected from the erythroid liquid culture at designed time points and resuspended with 200 μL of the culture medium. Cells were spun at 1350 rpm for 5 min onto glass slides using Cytospin™ 4 cytocentrifuge. The slides were stained with Leishman reagent according to the manufacturer’s instruction.

### Statistical analyses

Statistical analyses were performed using GraphPad Prism software. Data are expressed as mean ± SEM. Statistical significance was determined by Student’s *t* test or one-way ANOVA. *P* value of < 0.05 was considered statistically significant.

## Results

### GSK3 inhibitor promoted the efficient generation of mesoderm and hematoendothelial progenitors (HEPs)

Modulation of Wnt signaling has been shown to efficiently promote definitive mesoderm differentiation from hPSCs in vitro [7]. In this study, we used the GSK3 inhibitor CHIR99021 to activate Wnt signaling and induce mesoderm (ME) specification across our established iPSC lines, including the MUSIi001-A, MUSIi011-A, and PBiPSC1 cell lines (Fig. 1a). Upon differentiation, we noticed a lot of cell death during the first 2 days of differentiation; therefore, the medium was changed every day to remove dead cells. After 2 days of CHIR99021-induced differentiation, the number of differentiated iPSCs rapidly expanded up to 10-fold on day 3 and reached 100% confluence. We replated the differentiated cells onto a new culture vessel on day 3 and continued culturing the cells in the medium containing VEGF and FGF2 to promote cell expansion and differentiation toward HEPs. At this stage, the morphology of all the iPSC lines changed toward the endothelial-like cells (Fig. 1b). The expressions of sequential developmental markers were examined by flow cytometric analysis on days 3 and 5 of differentiation. Activation of the Wnt signaling pathway for 2 days induced all the three iPSC lines from undifferentiated cells (KDR^−^CD235a^−^CD34^−^) (Additional file 3: Figure S1a) toward mesodermal cells (KDR^+^CD235a^−^CD34^−^), which represented more than 60% of culture on day 3 of differentiation. The differentiated cells at this stage were negative for endothelial (CD31) (Fig. 1c) and HSPC markers (CD34, CD43, and CD45) (Additional file 3: Figure S1b). By day 5 of differentiation, approximately 80% of differentiated cells expressed CD34, CD31, and KDR, indicating that the cells were committed to the HEP stage (Fig. 1c). Importantly, we noticed that replating the day 3 differentiated cells onto new Matrigel®-coated plates enhanced the efficiency of HEP formation as compared to the non-replated culture, as evidenced by increased percentages of KDR^+^CD34^+^CD31^+^ HEPs on day 5 (Additional file 4: Figure S2). There was no significant difference in the percentages of mesodermal and HEP markers among the three iPSC lines (Fig. 1d). Our protocol demonstrated that CHIR99021 alone was sufficient to induce the formation of mesodermal cells and HEPs on day 3 and day 5, respectively. Supplementation with 5 ng/ml BMP4 did not increase the yield of mesodermal and HEP populations (Additional file 5: Figure S3).

We then analyzed the kinetics of the expression of primitive streak genes, *Brachyury* (*T*) and *KDR* on days 3 and 5 of differentiation. Activation of the Wnt signaling pathway by CHIR99021 resulted in downregulation of pluripotent genes, *OCT4* and *NANOG,* throughout the course of differentiation and upregulation of the mesendodermal marker, *Brachyury* (*T*), on day 3 of differentiation. In contrast, the expression of the mesodermal marker, *KDR*, increased with time (Fig. 1e). This data suggested that the induction of Wnt signaling by the GSK3 inhibitor CHIR99021 leads to the generation of HEPs through the formation of mesendodermal and mesodermal progenitors recapitulating the embryonic hematopoiesis [36].

### CHIR99021-induced HEPs gave rise to functional endothelial cells after being cultured in angiogenic condition

**Fig. 2:**
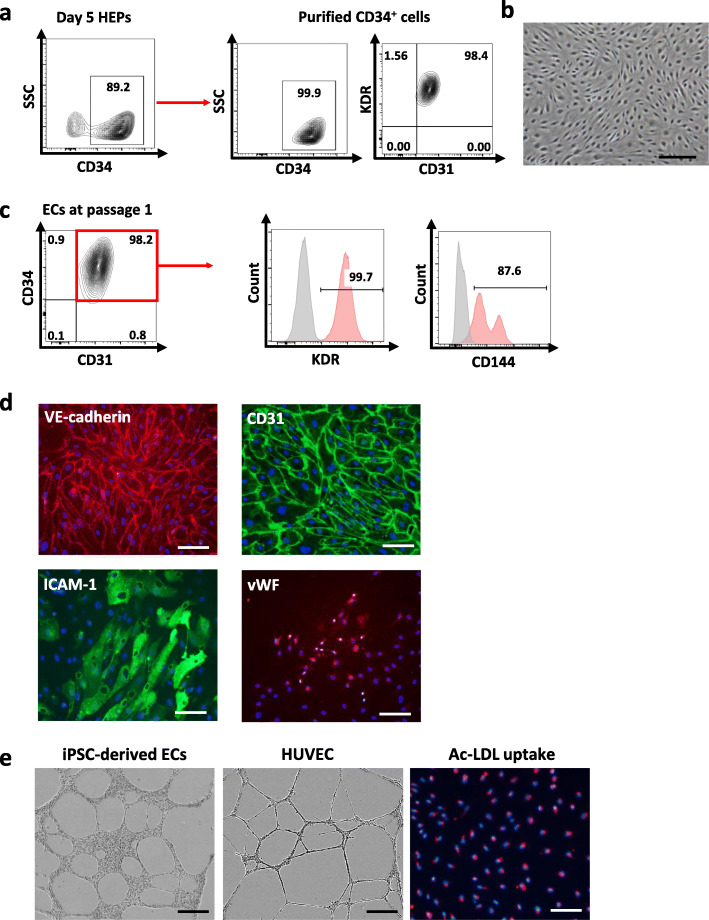
Characterization of ECs differentiated from the day 5 HEPs derived from the MUSIi011-A cells in endothelial growth medium (EGM). **a** CD34^+^ cells were isolated from the day 5 HEPs using a magnetic column and cultured in EGM for one passage. The purity of purified CD34^+^ cells after isolation was assessed by flow cytometric analysis. These CD34+ cells were 98% positive for KDR and CD31. **b** Morphology of ECs after culture in EGM for 1 passage (scale bar = 250 μm). **c** Flow cytometric result of endothelial markers, CD34, CD31, KDR, and CD144 (VE-cadherin). The CD34^+^CD31^+^ cells were gated for analysis of KDR and CD144. **d** Immunofluorescent staining for expression of VE-cadherin, CD31, ICAM-1, and vWF. Nuclei were counterstained with DAPI (scale bar = 100 μm). **e** Functional analysis of iPSC-derived ECs was performed using in vitro tube formation of capillary structures as compared to HUVECs (scale bar = 500 μm) and Ac-LDL uptake (scale bar = 100 μm)

To confirm whether the CHIR99021-induced HEPs have the potential to differentiate toward endothelial cells (ECs), we sorted CD34^+^ cells from day 5 HEPs derived from the MUSIi011-A line using a magnetic column. The purity of magnetic sorting was assessed by flow cytometry to ensure that more than 99% of the obtained cells were CD34^+^ cells. The sorted CD34^+^ cells were 98% positive for CD31 and KDR (Fig. 2a). This subpopulation was cultured on 0.1% gelatin-coated plates in the endothelial cell growth medium, which promoted the formation of mature ECs with homogeneous cobblestone morphology (Fig. 2b). These iPSC-derived ECs at passage 1 highly expressed CD34, CD31, KDR (VEGFR2), and CD144 (VE-cadherin) as analyzed by flow cytometry (Fig. 2c), and expressed late EC markers including adhesion molecules (CD31 and intercellular adhesion molecule 1 (ICAM-1)) and von Willebrand factor (vWF) as examined by immunofluorescent staining (Fig. 2d). We confirmed the functions of the iPSC-derived ECs by examining the ability to form a vascular-like network and the ability to uptake acetylated low-density lipoprotein (Ac-LDL). The iPSC-derived ECs formed vascular structures similar to those of HUVECs after being cultured on Matrigel® for 6 h. These cells were able to uptake Ac-LDL, which is indicative of endothelial function (Fig. 2e). Altogether, our data demonstrated that the HEPs were able to differentiate to functional ECs.

### Inhibition of TGF-β signaling promoted efficient endothelial-to-hematopoietic transition (EHT)

**Fig. 3:**
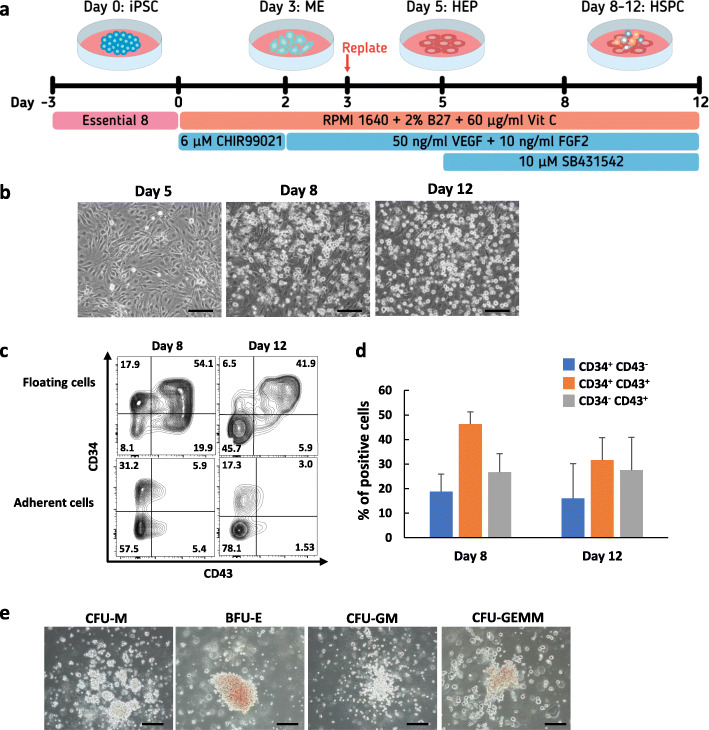
HSPC differentiation of the day 5 HEPs derived from the MUSIi011-A cells. **a** Schematic diagram of HSPC differentiation from iPSCs. **b** Representative images of differentiated cells on day 5 to day 12 (scale bar = 200 μm). **c** Representative flow cytometric results of HSPC markers, CD34, and CD43, at day 8 and day 12 of differentiation. **d** Percentages of subpopulations, CD34^+^CD43^−^, CD34^+^CD43^+^, and CD34^−^CD43^+^ cells at day 8 and day 12 of differentiation. Data are presented as mean ± SEM from five independent experiments. **e** Representative images of hematopoietic colonies from CFU assay. Day 8 floating cells were seeded in MethoCult™ medium for 2 weeks (scale bar = 200 μm)

To investigate whether the HEPs are capable of differentiating toward hematopoietic lineages. We further induced day 5 HEPs of the MUSIi011-A cell line derived from peripheral blood T lymphocytes, which carry rearranged T cell receptor (TCR) gene, toward HSPCs (Fig. 3a). Upon induction with the TGF-β inhibitor SB431542, we observed the emergence of floating cells from the adherent monolayer from day 8 onward. After collecting all the floating cells on day 8, we further cultured the adherent cells in the same medium for additional 4 days. The second wave of floating cells continuously emerged from the adherent monolayer (Fig. 3b). We closely monitored the EHT process using an Incucyte time-lapse microscope. The photographs were taken every hour for 3 days (from days 5 to 8 of differentiation). The round-shaped semi-adherent cells emerged from the endothelial-like cells within 12 h after supplementation with VEGF, FGF2, and SB431542. These cells formed apparent hematopoietic clusters as early as 24 h (Additional file 6: Movie S1). The hematopoietic-like cells on days 8 and 12 were harvested and analyzed by flow cytometry to confirm the expression of HSPC surface markers. Approximately 40–50% of floating cells were CD34^+^CD43^+^ cells whereas the adherent cells with endothelial-like morphology were negative for CD43 (Fig. 3c). Notably, the floating cells had CD34^+^CD43^−^, CD34^+^CD43^+^, or CD34^−^CD43^+^ phenotype indicating that we obtained a heterogeneous population of cells in different stages of hematopoietic development. The percentages of each subpopulation were not significantly different between days 8 and 12 samples (Fig. 3d). These floating cells from days 8 and 12 of differentiation were negative for CD45 (Additional file 7: Figure S4a). Further culture of the day 8 floating cells in HSPC expansion media, Stemline® II or StemPro™-34 medium, for a week gave rise to CD34^+^ CD45^+^ cells (Additional file 7: Figure S4b).

To assess the differentiation potential of HSPCs, we performed a CFU assay using the floating cells on day 8 of differentiation. The day 8 differentiated cells were able to generate multilineage hematopoietic colonies including CFU-M, BFU-E, CFU-GM and CFU-GEMM (Fig. 3e). However, most of the colonies represented granulocytes and macrophages (data not shown) indicating that day 8 HSPCs in our system had the myeloid-biased differentiation potential.

### CHIR99021-induced hematopoietic cells can give rise to the erythroid lineage

**Fig. 4:**
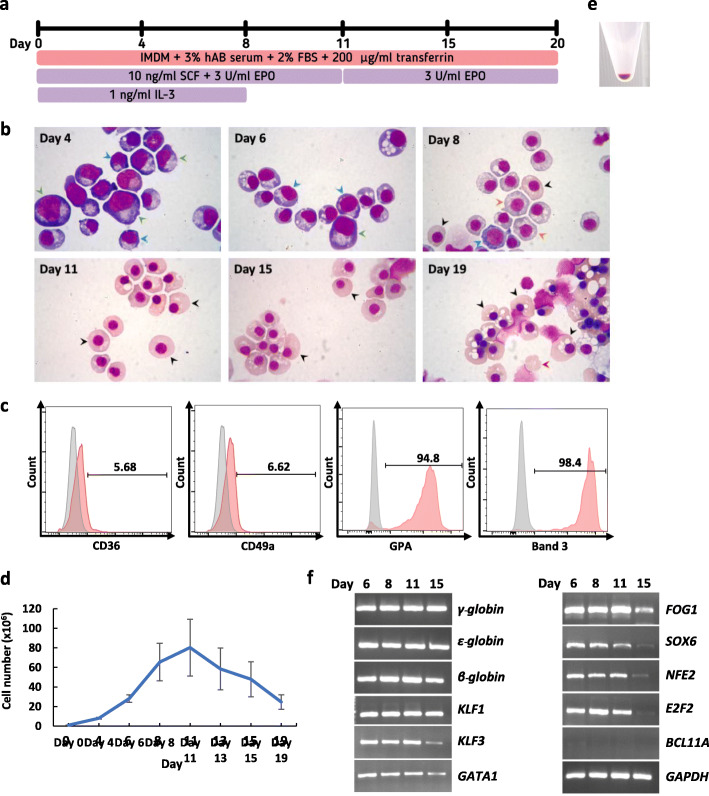
Erythroid differentiation of the day 8 HSPCs derived from the MUSIi011-A cells in the three-stage erythroid culture system. **a** Schematic diagram of the three-stage erythroid culture system. **b** Differentiated erythroid cells stained with Leishman reagent on days 4, 6, 8, 11, 15, and 19 of erythroid differentiation and analyzed by light microscopy. Green arrows represent proerythroblasts, blue arrows represent basophilic erythroblasts, orange arrows represent polychromatic erythroblasts, black arrows represent orthochromatic erythroblasts, and red arrows represent reticulocytes. **c** Flow cytometric analysis of erythroid markers, CD36, CD49a, GPA, and Band 3, at day 19 of erythroid differentiation. **d** Growth curve of differentiated cells from day 0 to 19. Error bars represent mean ± SEM of three independent experiments. **e** An image of cell pellet obtained on day 19 of erythroid differentiation. **f** Expression of erythroid-associated genes at days 6, 8, 11, and 15 by RT-PCR analysis

Differentiation potential toward T cells and erythroid cells expressing adult hemoglobin is indicative of definitive hematopoiesis [7]. In order to induce erythroid differentiation, the day 8 HSPCs were harvested and cultured in the established three-stage erythroid culture system (Fig. 4a). The HSPCs gradually converted to cells with morphology similar to those of proerythroblasts, basophilic erythroblasts, orthochromatic erythroblasts, and reticulocytes (Fig. 4b). Flow cytometric analysis was performed on day 19 cells and at least 95% of the cells expressed high levels of erythroid markers (GPA and Band 3), whereas less than 7% of the cells still expressed early erythroid markers (CD36 and CD49a) (Fig. 4c). The cells proliferated up to 25-fold at the end of the erythroid differentiation (Fig. 4d). The pellet of the differentiated cells on day 19 was red in color representing hemoglobin production (Fig. 4e). HPLC analysis showed that the cells produced predominantly fetal globin (54.6%) and embryonic globin (44.6%) with a small amount of adult globin (0.8%) (Additional file 8: Figure S5). RT-PCR analysis confirmed the expression of gamma globin, epsilon globin, and beta globin as well as other transcription factors known to be related to erythropoiesis including *KLF1, KLF3, GATA1, FOG1, SOX6, NFE2,* and *E2F2* on days 6, 8, 11, and 15 of erythroid differentiation (Fig. 4f). Interestingly, *BCL11A*, a transcription factor known to be a repressor of γ-globin expression [37, 38], was not detected in the differentiated cells. The absence of *BCL11A* expression may result in low adult globin levels in these cells. Taken together, these results demonstrated that the day 8 HSPCs were able to differentiate efficiently down the erythroid lineage.

### CHIR99021-induced HEPs and HSPCs can differentiate to T cell lineage with different properties

**Fig. 5:**
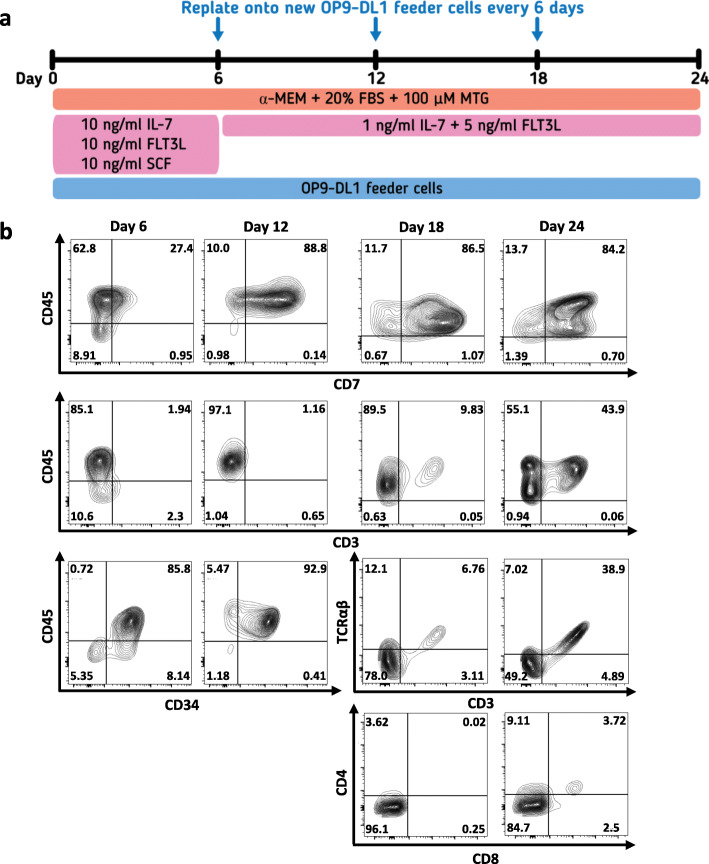
T cell differentiation of the day 5 HEPs derived from the MUSIi011-A cells in the OP9-DL1 co-culture system. **a** Schematic diagram of the OP9-DL1 co-culture system. The differentiated cells were seeded on the new OP9-DL1 cells every 6 days. The cells were harvested on day 24 for stimulation of TCR signaling. **b** Kinetic of T cell marker expression of the day 5 HEPs during the OP9-DL1 co-culture

We assessed the lymphoid potential of the iPSC-derived day 5 HEPs and day 8 HSPCs by co-culturing with the OP9-DL1 cells in the presence of SCF, FLT3L and IL-7 (Fig. 5a). The iPSC-derived HEPs developed to pro-T cell stage expressing CD34, CD45, and CD7 after co-culture with the OP9-DL1 cells for 12 days. At day 18 of co-culture, the differentiated cells started to express CD3 and TCRαβ. These cells were negative for both CD4 and CD8 indicating the commitment to double-negative (DN) T cell stage. We also observed the double-positive (DP) T cell (CD4^+^CD8^+^) population on day 24 of co-culture (Fig. 5b). In addition, we assessed the T cell differentiation potential of day 8 HSPCs using the same approach. The T cell differentiation pattern of the iPSC-derived HSPCs seemed to be earlier than those of the day 5 HEPs. The differentiated cells rapidly expressed early T cell lineage markers (CD45 and CD7) after co-culture with the OP9-DL1 cells for 6 days. The expression of CD3 was detected on day 12 and the DP T cell population appeared on day 18 of co-culture. However, the percentage of T cell lineage markers from the iPSC-derived day 8 HSPCs were not increased as much as those from the day 5 HEPs on day 24 of co-culture (Additional file 9: Figure S6a). In addition, the number of differentiated cells from the day 8 HSPCs was 9-fold lower compared to those from the day 5 HEPs (Additional file 9: Figure S6b).

**Fig. 6:**
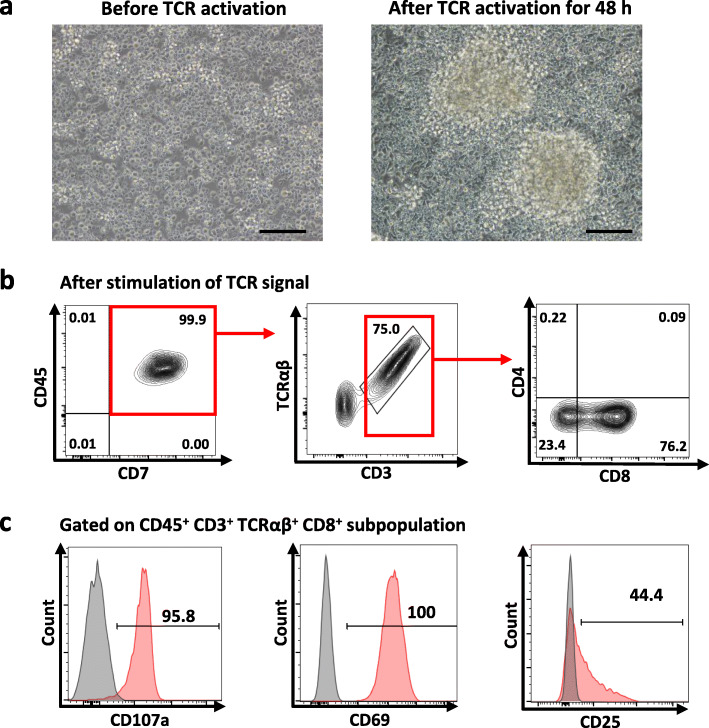
TCR stimulation after co-culture on the OP9-DL1 cells. **a** Morphology of the iPSC-derived T cells before and after stimulation with T Cell TransAct™ for 48 h. (scale bar = 200 μm). **b** Immunophenotype of mature T cells after TCR stimulation using T Cell TransAct™ for 2 weeks. **c** Flow cytometric analysis shows the expression of the early activation markers, CD107a and CD69, and the late activation marker, CD25, on the iPSC-derived T cells after TCR stimulation for 2 weeks. Gating was from mature T cell population (CD45^+^ CD3^+^ TCRαβ^+^ CD8^+^)

After day 24 of co-culture, single-positive (SP) mature T cells were induced by TCR stimulation. The differentiated T cells from day 5 HEPs were activated with T Cell TransAct™ (anti-CD3/CD28) (Fig. 6) or anti-CD3 (Additional file 10: Figure S7a) for 14 days. TCR stimulation for 48 h resulted in morphological changes from single cells to multiple clusters (Fig. 6a) and the cells proliferated up to 34-fold and 30-fold for T Cell TransAct™ and anti-CD3 treatments, respectively (Additional file 10: Figure S7b). The differentiated cells developed to mature CD8 SP T cells with the phenotype of CD45^+^ CD7^+^CD3^+^TCRαβ^+^CD4^−^CD8^+^ (Fig. 6b). We also assessed the T cell response after TCR stimulation by flow cytometry. The majority of mature CD8 SP T cells expressed the early activation markers, CD107a and CD69, and approximately 44% of the SP T cells expressed the late activation marker, CD25, after TCR activation (Fig. 6c). These data suggested that activation with anti-CD3 antibody or T Cell TransAct™ is sufficient to induce SP specification of the iPSC-derived T cells, which exhibited the T cell function by responding to TCR activation in terms of cell proliferation and expression of activation markers.

## Discussion

iPSC technology offers potential cell-based therapy for patients with diverse hematological diseases. However, applications of iPSC-derived hematopoietic cells are often restricted by differentiation protocols to generate large numbers of HSPCs and terminally differentiated hematopoietic cells in a robust manner. In addition, cell products are quite variable in terms of quantity, quality, and differentiation stages. These variations limit the use of iPSC-derived HSPCs for in vitro and in vivo experiments, drug screening, and possible future clinical applications.

Our study describes a monolayer serum-free and feeder-free protocol to efficiently generate HEPs from hiPSCs. Our approach is relatively simple in terms of differentiation procedure and cytokine usage. We obtained high percentages of the KDR^+^CD34^+^CD31^+^ population on day 5 of differentiation compared to the previous protocols [24, 31]. Using the defined medium and low starting cell number, we obtained reproducible results from three iPSC lines derived from different somatic cell sources. Modulation of Wnt-β-catenin by the GSK3 inhibitor CHIR99021 specified hiPSCs to hematopoietic lineage as determined by the appearance of KDR^+^CD235a^−^ mesoderm, the early precursors of definitive hematopoiesis in the hPSC differentiation system [7, 22]. Our results demonstrated that the use of 6 μM CHIR99021 alone can induce mesodermal differentiation. These data were consistent with previous reports [23, 31, 39]. However, Patsch et al. demonstrated that Wnt activation by CHIR99021 at concentrations of 6–10 μM required BMP4 supplementation to initiate mesodermal commitment [40], suggesting that different basal medium composition may lead to variation in differentiation efficiency. Upon exposure to VEGF and FGF2, ~ 60% of differentiated cells were positive for KDR, CD34, and CD31, referred to as HEPs. Previous reports indicated that HEPs have differentiation potential toward hematopoietic and endothelial lineages [13, 41–43]. In this study, we confirmed that HEPs exhibited endothelial differentiation potential in the endothelial growth medium. After culturing for one passage, we observed homogeneous monolayer of cells showing endothelial characteristics including expression of VE-cadherin, ICAM-1, and vWF together with the ability to form vascular-like network and uptake Ac-LDL. In addition, we demonstrated that the HEPs display HSPC differentiation potential by supplementing the culture medium with the TGF-β inhibitor SB431542 to induce the EHT process. The emergence of hematopoietic cells from the endothelial monolayer was observed within 24 h. These cells have multilineage differentiation potential to give rise to all types of hematopoietic colonies in the CFU assay. Although day 8 HSPCs contain a heterogeneous population as determined by the expression of CD34 and CD43, most of the cells (~ 50%) were CD34^+^CD43^+^. In agreement with the previous reports [4, 44], we found that the expression of CD43 was restricted to hematopoietic-like cells, suggesting that CD43 is the initial marker of HSPCs that emerged from the HEPs. We did not observe the expression of CD45 in the day 8 or day 12 HSPCs. This result was consistent with the previous observation that CD43 expression was detected in the earliest hematopoietic progenitor cells prior to the onset of CD45 and CD41 expression during hematopoietic differentiation from hPSCs [44, 45]. However, a prolonged culture of the day 8 HSPCs in HSPC expansion media or co-culture with the OP9-DL1 cells resulted in the emergence of the CD45-positive population, which can be differentiated into more mature blood cells such as T cells.

We investigated the erythroid differentiation potential of the day 8 HSPCs using an erythroid liquid culture system. The day 8 HSPCs were efficiently differentiated to erythroid cells expressing γ-, ε-, and low levels of β-globin with a small proportion of the cells enucleated and became reticulocytes. The obtained erythroid cells were similar to those from previous studies, which does not represent adult red cell phenotype [46–48]. In this study, we detected the expression of various transcription factors known to be related to erythropoiesis during erythroid differentiation; however, *BCL11A* was not expressed throughout the differentiation. Previous studies demonstrated that BCL11A is the key regulator of fetal to adult globin switching by repressing γ-globin expression [37, 38]. Ectopic expression of KLF1 and BCL11A-XL in the iPSC-derived erythroid cell line and cord blood-derived erythroid cells resulted in an increase in the β-globin production to adult levels and decrease in the ε- and ζ-globin production [38]. The absence of *BCL11A* expression may result in low adult globin levels in our system. Therefore, induction of BCL11A expression in these iPSC-derived erythroid cells may alter the fetal/embryonic phenotype of these cells to the adult phenotype. Further optimization is required to obtain more efficient red blood cell production before these cells can be applied for therapeutic use.

During embryonic development, the two hematopoietic waves are distinguishable; however, this is not the case for the in vitro hematopoietic differentiation system. Once HSPCs are generated in the system, the primitive and definitive HSPCs cannot be distinguished by differential surface markers; therefore, T lymphocyte potential has been used as one of the indicators for the specification of the definitive hematopoiesis [7, 19, 49]. Generation of T lymphocytes from iPSCs is a complex process and requires tightly regulated signals recapitulating developmental ontogeny of T lymphopoiesis, which mainly involves two essential stages; iPSCs require appropriate signals from microenvironments for definitive HSC commitment followed by Notch signaling for T cell development and maturation [50]. To confirm that our system generated HSPCs having lymphoid potential, we performed T lymphocyte differentiation by transferring the day 5 HEPs and day 8 HSPCs onto the OP9 cells overexpressing Notch ligand, Delta-like 1 (OP9-DL1) in the presence of Flt3L, SCF and IL-7 for further differentiation into pro-T cells, which later require TCR signal to become mature SP T cells [51]. Our data demonstrated that the day 5 HEPs and day 8 HSPCs were able to generate CD45^+^ CD7^+^ CD4^+^ CD8^+^ CD3^+^ TCRαβ^+^ T cells after co-culture with the OP9-DL1 cells indicating acquisition of the definitive hematopoietic program. Furthermore, after TCR stimulation for 14 days, mature CD8 SP T cells appeared from the day 5 HEP population. Importantly, the day 8 HSPCs were differentiated toward T cell lineage faster than the day 5 HEPs. It is possible that day 8 HSPCs were directly committed to T cell lineage after co-culture with the OP9-DL1 cells whereas T cell specification of the day 5 HEPs was delayed due to the requirement of EHT process during the OP9-DL1 co-culture. However, we found that the proliferation capacity of T cells during T cell differentiation was considerably robust in the day 5 HEPs compared to that of the day 8 HSPCs. Similarly, Kumar et al. demonstrated that hematovascular mesodermal progenitors (HVMPs), which expressed KDR^hi^CD31^−^ from the culture of day 4 in the OP9 co-culture system, produced T cells with robust expansion as compared to the more established multipotent hematopoietic progenitors (MHP), which produced fewer progenitor T cells with limited proliferation [21].

Notch signaling has been known as a critical signal for the specification of definitive hematopoiesis from hiPSCs [21, 49, 52, 53]. Activation of Notch signaling during an early stage specifies arterial HE, which is highly enriched in lympho-myeloid progenitors. Therefore, the day 5 HEPs may undergo arterial HE specification during co-culture with the OP9-DL1 cells, leading to robust T cell production. On the other hand, activation of Notch signaling during the day 8 HSPCs would bypass the enhancing effect on arterial HE generation leading to the inefficient production of T cells [21]. Recently, several studies demonstrated that hPSC-derived T lymphocytes have therapeutic potential for adoptive cell therapy (ACT) to treat patients with cancers [5, 54–56], viral diseases [25], and regaining self-tolerance by delivering regulatory T cells [57, 58]. Our differentiation strategy in combination with genome editing of T cell receptor (TCR) and chimeric antigen receptor (CAR) technology may facilitate translational medicine by providing scalable production of off-the-shelf universal iPSC-derived T cell products for ACT [59–61].

## Conclusions

This study established a feeder-free and serum-free monolayer differentiation method to derive multilineage HEPs, which are precursors of endothelium and hematopoietic cells including erythroid and T lymphoid cells. This protocol provides a simple, efficient, and less expensive approach to generate hematopoietic cells. Our approach will be instrumental in studying hematopoiesis in vitro. In addition, this method also can be useful for generating a large number of hematopoietic cells for both fundamental research and regenerative medicine in the future.

## Supplementary Information

**Additional file 1:** **Table S1.** Primer sequences for qPCR.

**Additional file 2:** **Table S2.** Primer sequences for RT-PCR.

**Additional file 3:** **Figure S1.** Differentiation of iPSCs toward mesodermal cells. **a** Representative flow cytometric analysis shows that day 0 undifferentiated cells did not express KDR, CD235a, CD34 and CD31. **b** Representative flow cytometric analysis shows that on day 3 of differentiation, the KDR+CD235a- mesodermal population did not express hematopoietic and endothelial markers.

**Additional file 4:** **Figure S2.** Differentiation of iPSCs toward HEPs. **a** Morphology of the differentiated MUSIi011-A cells on day 5 in replating and non-replating conditions. Scale bar = 200 μm. **b** Flow cytometric analysis shows the expression of HEP markers on day 5 of differentiation. The KDR^+^CD235a^−^ cells were gated for analysis of the HEP markers (KDR^+^CD34^+^CD31^+^).

**Additional file 5:** **Figure S3.** Differentiation of iPSCs toward HEPs in the presence of BMP4. On day 0, the medium was supplemented with 5 ng/mL BMP4. On days 1 and 2, the medium was supplemented with 5 ng/mL BMP4 and 2 µM CHIR99021. **a** Morphology of the differentiated MUSIi011-A cells on day 3 and day 5 in the condition with BMP4 supplementation. Scale bar = 200 μm. **b** Flow cytometric analysis shows the expression of HEP markers at days 3 and 5 of differentiation. The KDR^+^CD235a^−^ cells were gated for analysis of the HEP markers (KDR^+^CD34^+^CD31^+^).

**Additional file 6:** **Movie S1.** Formation of hematopoietic-like cells from days 5 to 8 of differentiation.

**Additional file 7:** **Figure S4. a** Flow cytometric analysis shows the expression of CD34 and CD45 of day 8 and day 12 floating cells. **b** Day 8 floating cells were harvested and transferred to culture in HSPC expansion mediums: Stemline® II or StemPro™-34 for 7 days. Flow cytometric analysis shows the expression of HSPC markers: CD34 and CD45 after culture for 4 and 7 days.

**Additional file 8:** **Figure S5.** HPLC trace shows fetal (F), adult and embryonic hemoglobin levels in the erythroid cells differentiated from the MUSIi011-A cells.

**Additional file 9:** **Figure S6.** T cell differentiation in the OP9-DL1 co-culture system. **a** Kinetic of T cell marker expression of the day 8 HSPCs during the OP9-DL1 co-culture. **b** Growth curve of differentiated cells from the day 5 HEPs and the day 8 HSPCs during the OP9-DL1 co-culture.

**Additional file 10:** **Figure S7.** TCR stimulation after co-culture on the OP9-DL1 cells. **a** Immunophenotype of mature T cells after TCR stimulation using anti-CD3 antibody and without TCR activator after 2 weeks of culture. **b** Number of total cells upon TCR activation using T cell TransAct™ or anti-CD3 antibody.

## Data Availability

All data generated or analyzed during this study are included in this published article (and its supplementary information files).

## Abbreviations

ACT: Adoptive cell therapy
Ac-LDL: Acetylated low-density lipoprotein
AGM: Aorta-gonad-mesonephros
BMP4: Bone morphogenetic protein 4
CAR: Chimeric antigen receptor
CFU: Colony-forming unit
DN: Double-negative
DP: Double-positive
EBs: Embryoid bodies
ECs: Endothelial cells
EHT: Endothelial-to-hematopoietic transition
FBS: Fetal bovine serum
FGF2: Fibroblast growth factor 2
HEPs: Hematoendothelial progenitors
hiPSCs: Human induced pluripotent stem cells
hPSCs: Human pluripotent stem cells
HSCs: Hematopoietic stem cells
HSPCs: Hematopoietic stem/progenitor cells
HUVECs: Human umbilical vein endothelial cells
HVMPs: Hematovascular mesodermal progenitors
ICAM-1: Intercellular adhesion molecule 1
ME: Mesoderm
MHP: Multipotent hematopoietic progenitors
SP: Single-positive
TCR: T cell receptor
VEGF: Vascular endothelial growth factor
vWF: Von Willebrand factor
