# Supplementary material for: Multilineage differentiation potential of hematoendothelial progenitors derived from human induced pluripotent stem cells
Source: Stem Cell Res Ther. 2020 Nov 11;11:481. doi: 10.1186/s13287-020-01997-w (PMC7659123; doi:10.1186/s13287-020-01997-w)
Supplement: Supplementary file 1 — Additional file 1: Table S1. Primer sequences for qPCR. [file 13287_2020_1997_MOESM1_ESM.doc]

**Table S1. Primer sequences for qPCR (related to Experimental Procedures)**

| **Target** | **Primer sequence** | **Product size** |
| --- | --- | --- |
| *OCT4* | Forward: TCG AGA ACC GAG TGA GAG G  Reverse: GAA CCA CAC TCG GAC CAC A | 125 bp |
| *NANOG* | Forward: AGA TGC CTC ACA CGG AGA CT  Reverse: GGA CTG GTG GAA GAA TCA GG | 85 bp |
| *Brachyury* | Forward: GCT GTG ACA GGT ACC CAA CC  Reverse: CAT GCA GGT GAG TTG TCA GAA | 119 bp |
| *KDR* | Forward: TGA GCA AAG GGT GGA GGT GAC T  Reverse: CTT GCAC AAA GTG ACA CGT TGA G | 90 bp |
| *GAPDH* | Forward: GTC AAC GGA TTT GGT CGT ATT G  Reverse: CAT GGG TGG AAT CAT ATT GGA A | 180 bp |
