# Supplementary material for: Multilineage differentiation potential of hematoendothelial progenitors derived from human induced pluripotent stem cells
Source: Stem Cell Res Ther. 2020 Nov 11;11:481. doi: 10.1186/s13287-020-01997-w (PMC7659123; doi:10.1186/s13287-020-01997-w)
Supplement: Supplementary file 2 — Additional file 2: Table S2. Primer sequences for RT-PCR. [file 13287_2020_1997_MOESM2_ESM.doc]

**Table S2. Primer sequences for RT-PCR (related to Experimental Procedures)**

| **Target** | **Primer sequence** |
| --- | --- |
| Gamma globin | Forward: 5’- TGGGTCATTTCACAGAGGAGR  Reverse : 5’-AGACAACCAGGAGCCTTCC |
| Epsilon globin | Forward: 5’- TTTTACTGCTGAGGAGAAGGCTGCC  Reverse: 5’- CTTGCCAAAGTGAGTAGCCAGAATAA |
| Beta globin | Forward: 5’- GCTCACCTGGACAACCTCAR  Reverse : 5’- CGTTGCCCAGGAGCCTGAA |
| *KLF1* | Forward: 5′-cgaagagctacaccaagagc  Reverse: 5′-gctgtctatgggtccgtgtt |
| *KLF3* | Forward: 5′-ctactccacaccattgcctgag  Reverse: 5′-cacgatgaccgaagggtgattc |
| *GATA1* | Forward: 5′-ttcagcagcctattcctctcc  Reverse: 5′-ccttggtagagatgggcagta |
| *FOG1* | Forward: 5′-aaggacaggaaccagaacccag  Reverse: 5′-ctctgctggctccttcttca |
| *SOX6* | Forward: 5′-tgatggagaggatgcaatgacc  Reverse: 5′-ccatttgctgccgttgtttct |
| *NFE2* | Forward: 5′-gagatggaactgacttggcagg  Reverse: 5′-gcacttccagtctcgtctgcg |
| *E2F2* | Forward: 5′-ctcggtatgacacttcgctgg  Reverse: 5′-gcacttccagtctcgtctgcg |
| *BCL11A* | Forward: 5′-tcacgccagaggatgacgatt  Reverse: 5′-tcaagtgatgtctcggtggtgg |
| *GAPDH* | Forward: 5’-ACCACAGTCCATGCCATCAC  Reverse: 5’-TCCACCACCCTGTTGCTGTA |
