# Supplementary figures and images for: Multilineage differentiation potential of hematoendothelial progenitors derived from human induced pluripotent stem cells
Source: Stem Cell Res Ther. 2020 Nov 11;11:481. doi: 10.1186/s13287-020-01997-w (PMC7659123; doi:10.1186/s13287-020-01997-w)

Figure S1

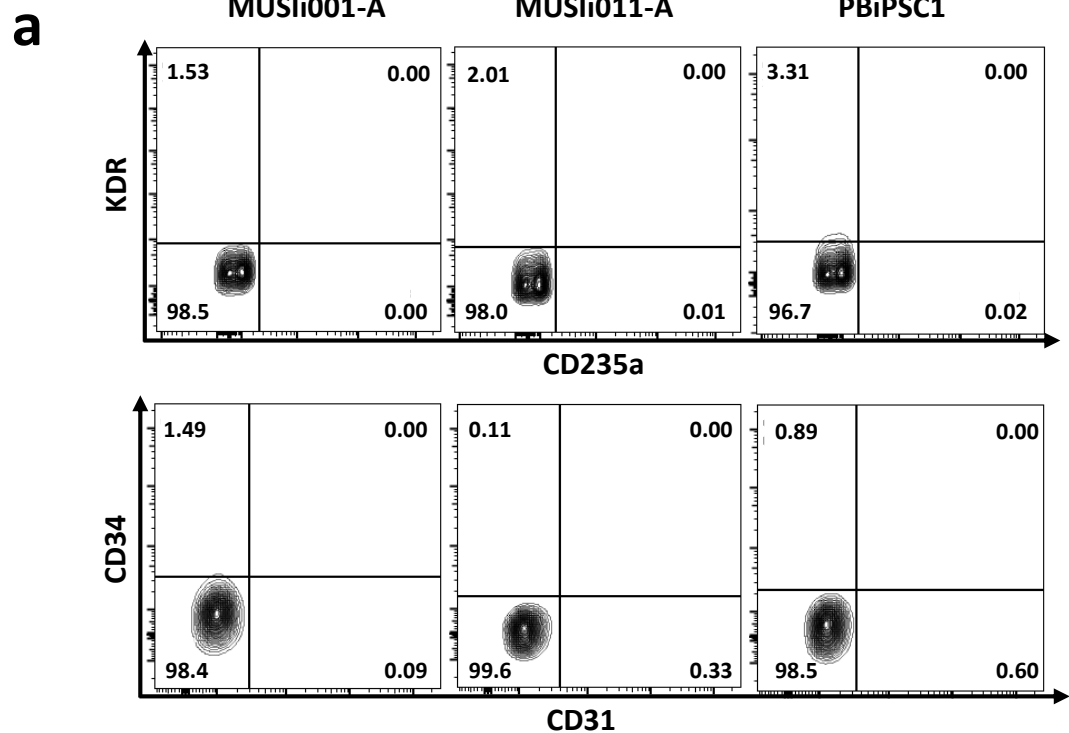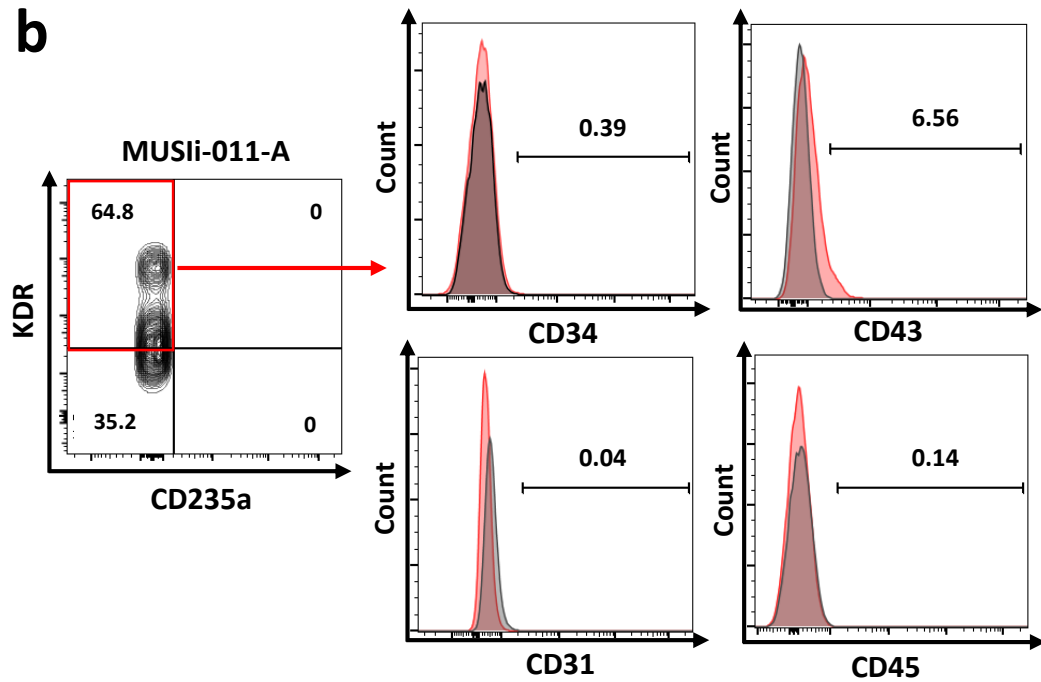

Supplement: Supplementary file 3 — Additional file 3: Figure S1. Differentiation of iPSCs toward mesodermal cells. a Representative flow cytometric analysis shows that day 0 undifferentiated cells did not express KDR, CD235a, CD34 and CD31. b Representative flow cytometric analysis shows that on day 3 of differentiation, the KDR+CD235a- mesodermal population did not express hematopoietic and endothelial markers. [file 13287_2020_1997_MOESM3_ESM.pdf]

Figure S2

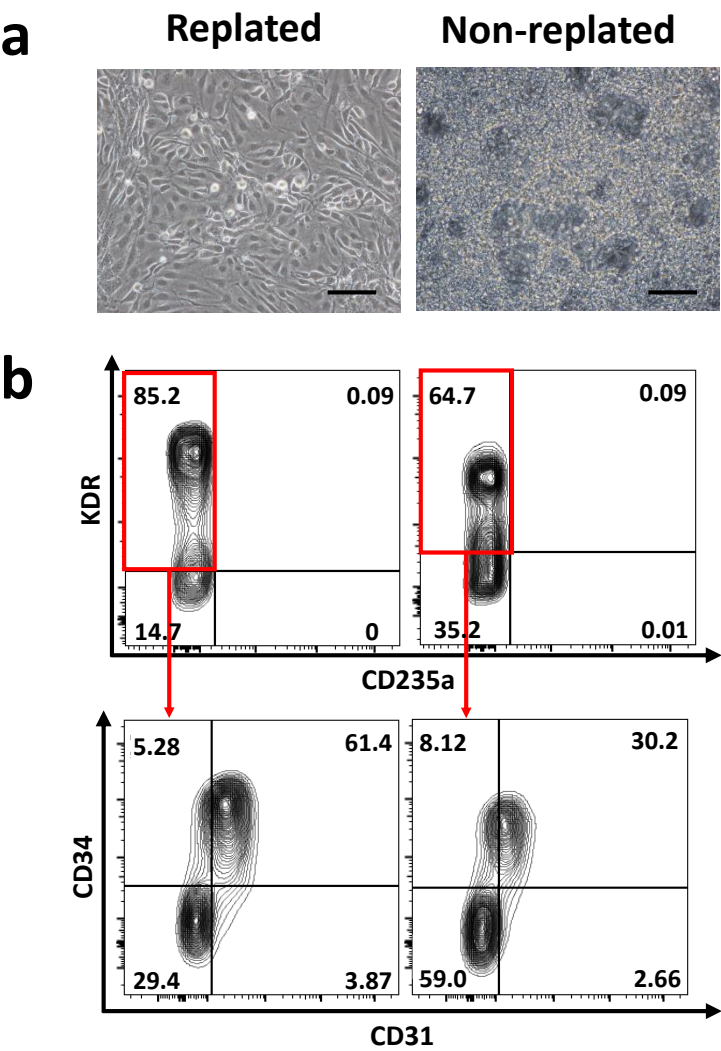

Supplement: Supplementary file 4 — Additional file 4: Figure S2. Differentiation of iPSCs toward HEPs. a Morphology of the differentiated MUSIi011-A cells on day 5 in replating and non-replating conditions. Scale bar = 200 μm. b Flow cytometric analysis shows the expression of HEP markers on day 5 of differentiation. The KDR+CD235a− cells were gated for analysis of the HEP markers (KDR+CD34+CD31+). [file 13287_2020_1997_MOESM4_ESM.pdf]

Figure S3

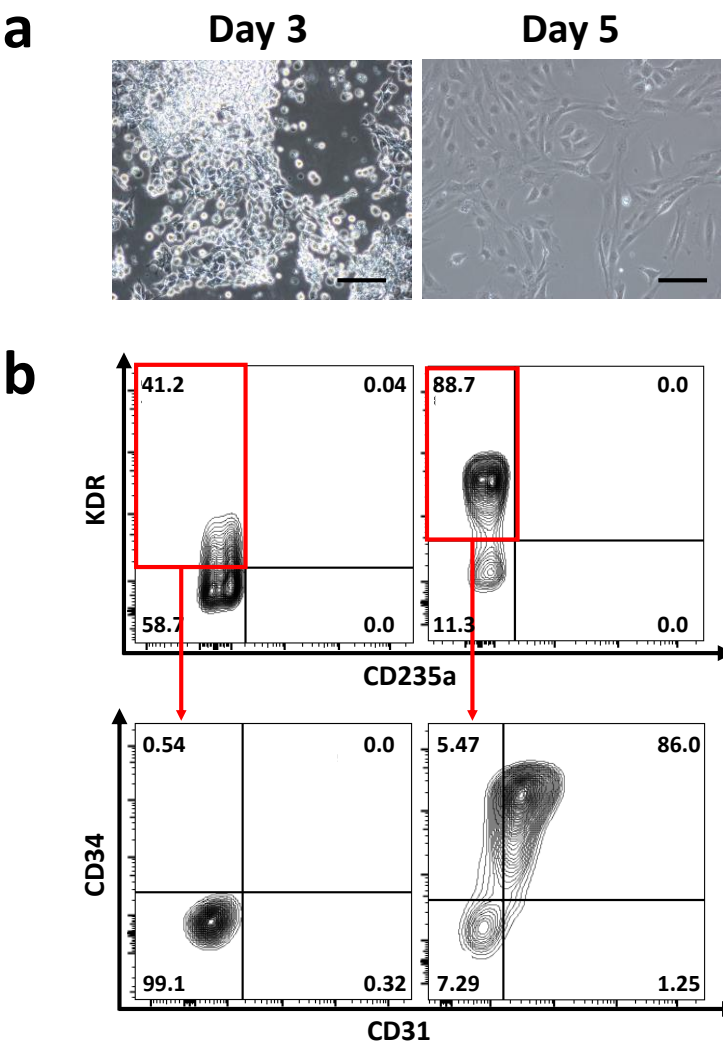

Supplement: Supplementary file 5 — Additional file 5: Figure S3. Differentiation of iPSCs toward HEPs in the presence of BMP4. On day 0, the medium was supplemented with 5 ng/mL BMP4. On days 1 and 2, the medium was supplemented with 5 ng/mL BMP4 and 2 µM CHIR99021. a Morphology of the differentiated MUSIi011-A cells on day 3 and day 5 in the condition with BMP4 supplementation. Scale bar = 200 μm. b Flow cytometric analysis shows the expression of HEP markers at days 3 and 5 of differentiation. The KDR+CD235a− cells were gated for analysis of the HEP markers (KDR+CD34+CD31+). [file 13287_2020_1997_MOESM5_ESM.pdf]

Figure S4

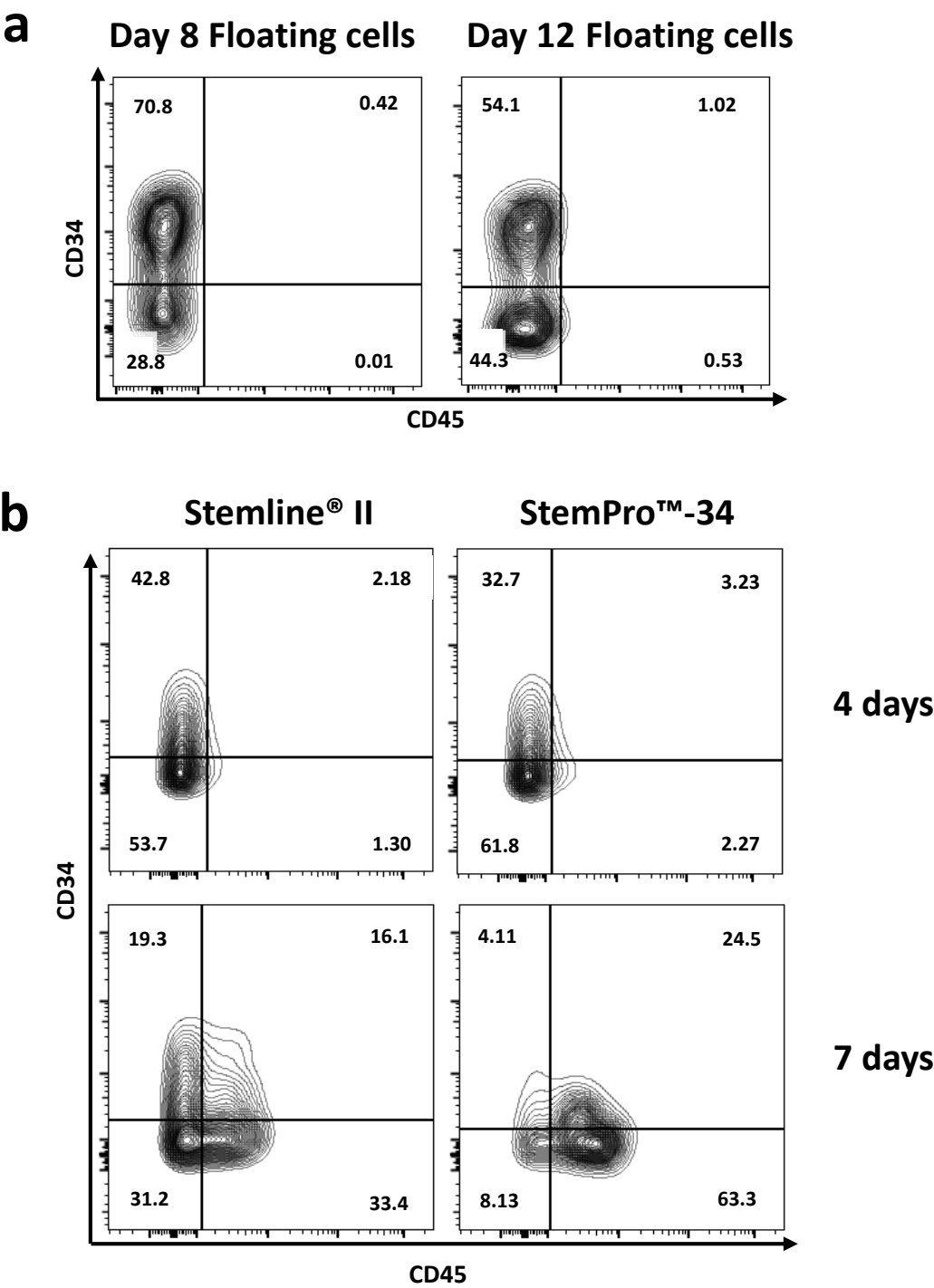

Supplement: Supplementary file 7 — Additional file 7: Figure S4. a Flow cytometric analysis shows the expression of CD34 and CD45 of day 8 and day 12 floating cells. b Day 8 floating cells were harvested and transferred to culture in HSPC expansion mediums: Stemline® II or StemPro™-34 for 7 days. Flow cytometric analysis shows the expression of HSPC markers: CD34 and CD45 after culture for 4 and 7 days. [file 13287_2020_1997_MOESM7_ESM.pdf]

Figure S5

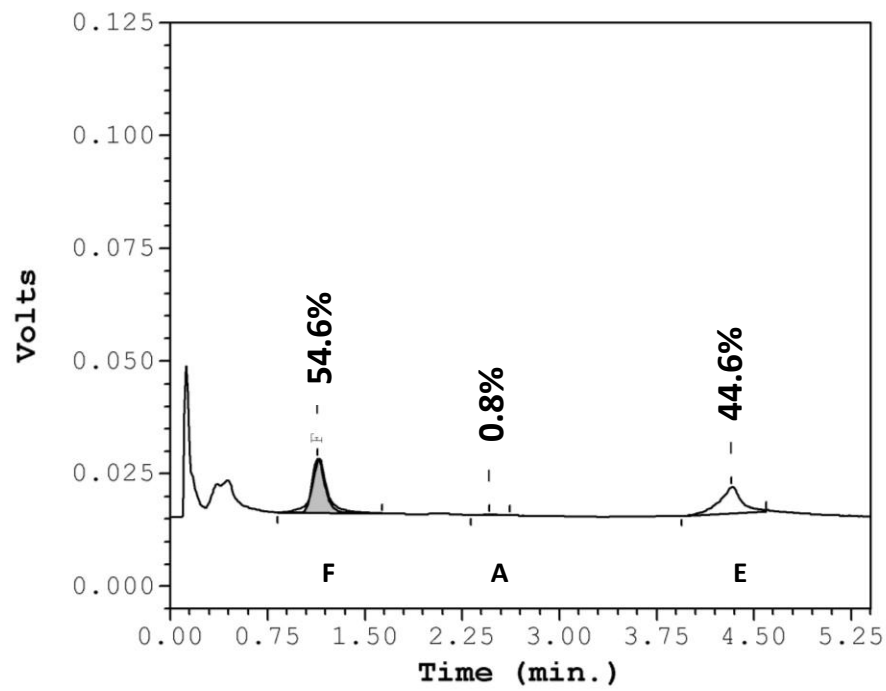

Supplement: Supplementary file 8 — Additional file 8: Figure S5. HPLC trace shows fetal (F), adult and embryonic hemoglobin levels in the erythroid cells differentiated from the MUSIi011-A cells. [file 13287_2020_1997_MOESM8_ESM.pdf]

Figure S6

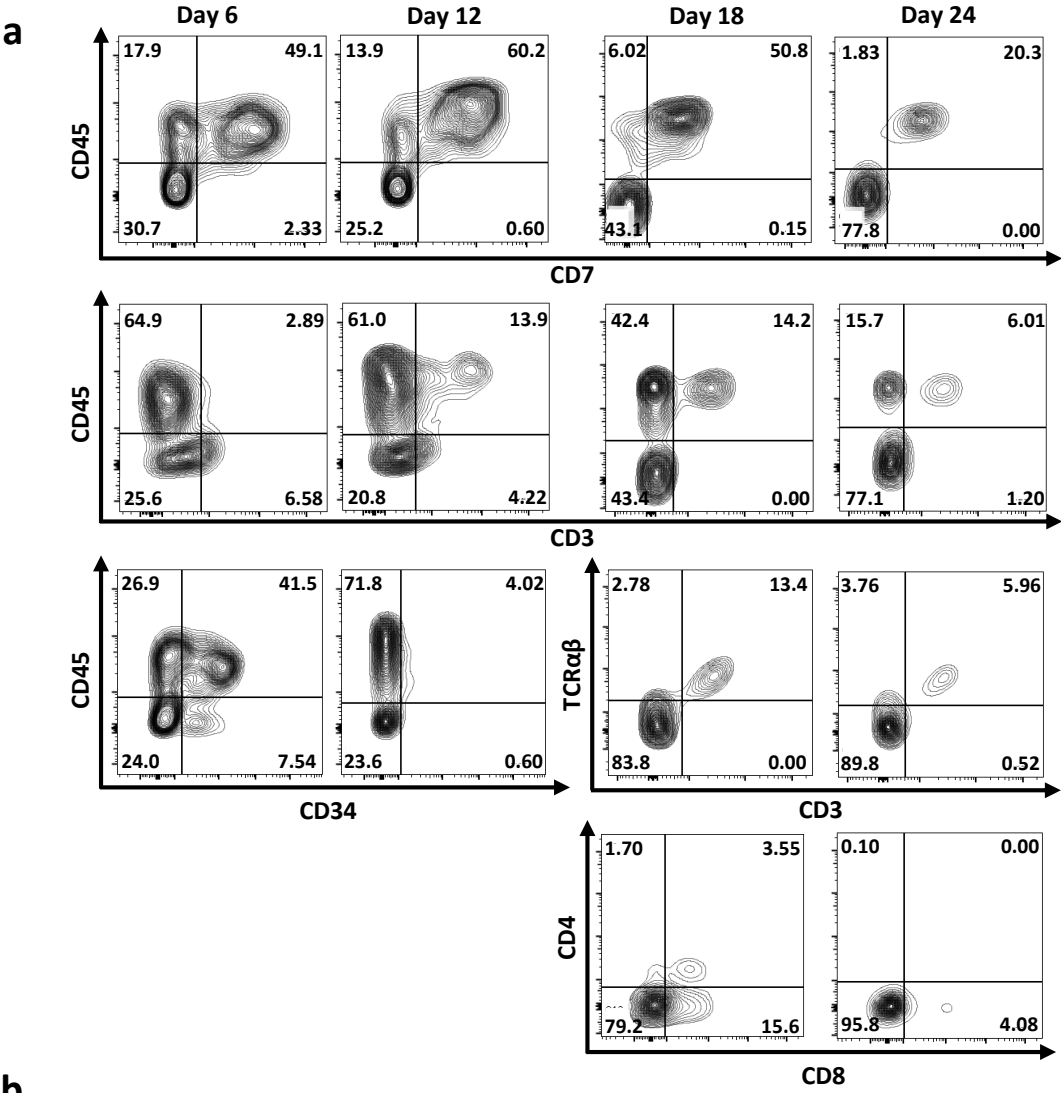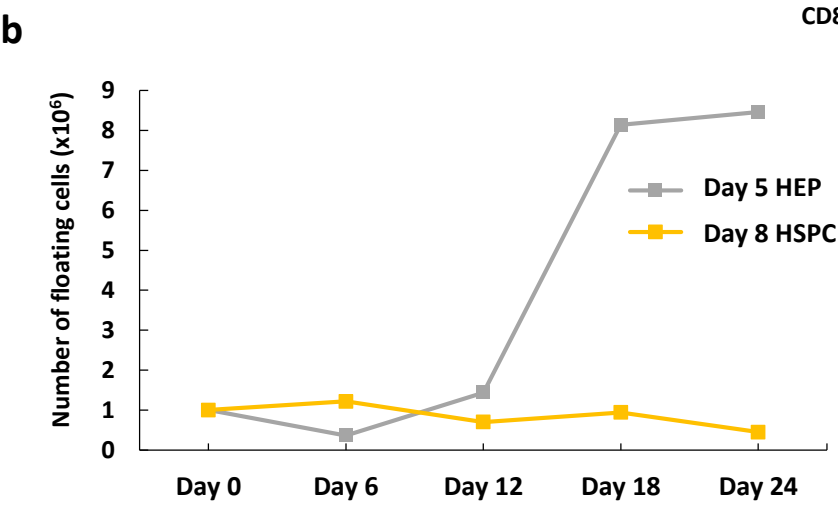

Supplement: Supplementary file 9 — Additional file 9: Figure S6. T cell differentiation in the OP9-DL1 co-culture system. a Kinetic of T cell marker expression of the day 8 HSPCs during the OP9-DL1 co-culture. b Growth curve of differentiated cells from the day 5 HEPs and the day 8 HSPCs during the OP9-DL1 co-culture. [file 13287_2020_1997_MOESM9_ESM.pdf]

Figure S7

a

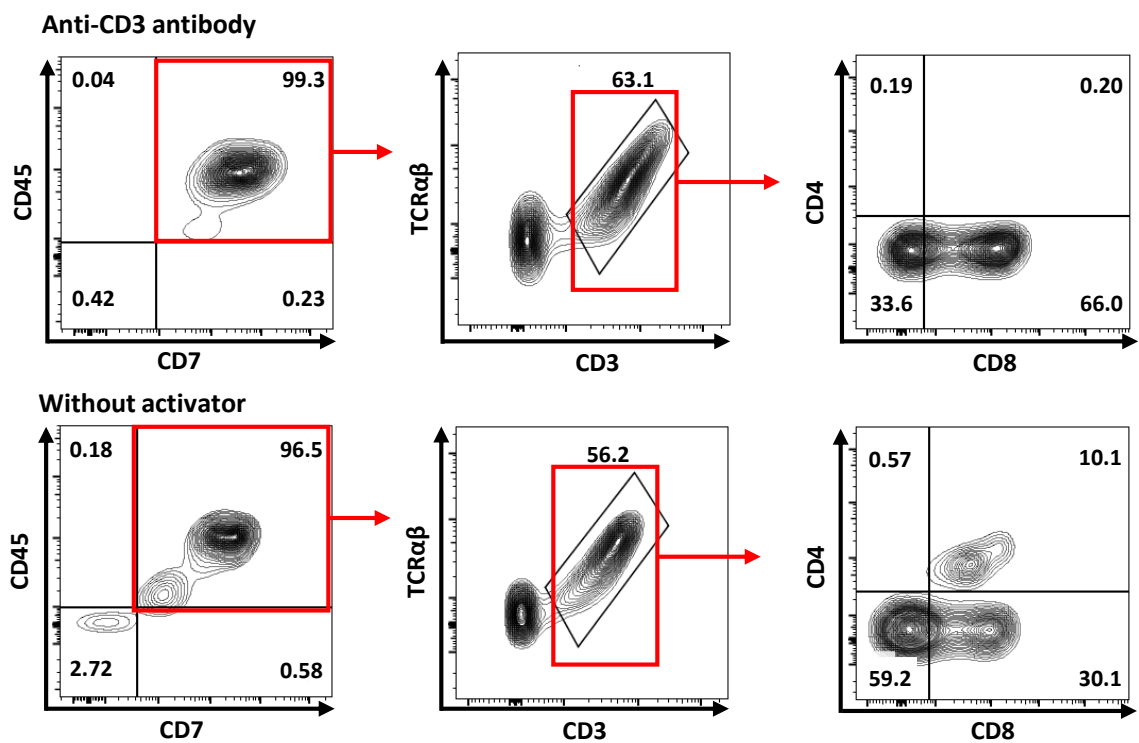

b

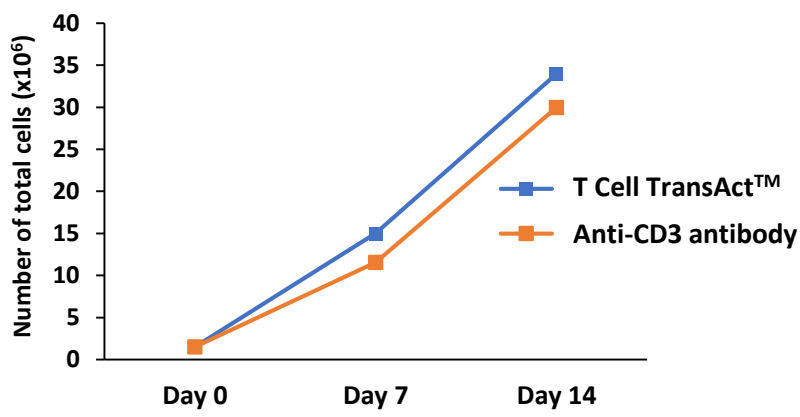

Supplement: Supplementary file 10 — Additional file 10: Figure S7. TCR stimulation after co-culture on the OP9-DL1 cells. a Immunophenotype of mature T cells after TCR stimulation using anti-CD3 antibody and without TCR activator after 2 weeks of culture. b Number of total cells upon TCR activation using T cell TransAct™ or anti-CD3 antibody. [file 13287_2020_1997_MOESM10_ESM.pdf]
